# High Prevalence of Anal Oncogenic Human Papillomavirus Infection in Young Men Who Have Sex with Men Living in Bamako, Mali

**DOI:** 10.1186/s13027-021-00385-0

**Published:** 2021-07-01

**Authors:** Donato Koyalta, Ralph-Sydney Mboumba Bouassa, Almoustapha Maiga, Aliou Balde, Jules Bashi Bagendabanga, Almahdy Ag Alinity, David Veyer, Hélène Péré, Laurent Bélec

**Affiliations:** 1 Centre Hospitalo-Universitaire Gabriel Touré, Bamako Bamako, Mali; 2 Faculté des Sciences de la Santé Humaine de N’Djamena, N’Djamena, Chad; 3 Ecole Doctorale Régionale en Infectiologie Tropicale, Franceville, Gabon; 4 Laboratoire de Virologie, Hôpital Européen Georges Pompidou, Assistance Publique-Hôpitaux de Paris, and Université de Paris, Paris, France; 5 Laboratoire du Centre Hospitalo-Universitaire Gabriel Touré, Bamako, Mali; 6 Pierre Louis Institute of Epidemiology and Public Health (IPLESP), Sorbonne University, INSERM, Paris, France; 7 Project Linkages – Family Health International, Bamako, Mali; 8 Clinique Soutoura, Bamako, Mali

**Keywords:** HPV, Anal, Molecular epidemiology, MSM, Prophylactic vaccine, Gardasil, Mali

## Abstract

**Background:**

High-risk human papillomavirus (HR-HPV) anal infection is a major problem among men who have sex with men (MSM) living in sub-Saharan Africa. The prevalence of anal HR-HPV infection and associated risk factors were estimated in a cross-sectional study in MSM living in Bamako, Mali.

**Methods:**

MSM consulting at sexual health center of the National NGO Soutoura, Bamako, were prospectively included. Sociodemographic and clinical-biological data were collected. HPV detection and genotyping were performed from anal swabs using multiplex real-time PCR. Risk factors associated with anal HPV infection were assessed by logistic regression analysis.

**Results:**

Fifty MSM (mean age, 24.2 years; range, 18–35) of which 32.0% were infected with HIV-1, were prospectively included. The overall prevalence of anal HPV infection of any genotypes was 70.0% (35/50) with 80.0% (28/35) of swabs positive for HR-HPV. HR-HPV-58 was the most detected genotype [13/35 (37.1%)], followed by HR-HPV-16 and low-risk (LR)-HPV-6 [12/35 (34.2%)], LR-HPV-40 [10/35 (28.6%)], LR-HPV-11 [9/35 (25.7%)], HR-HPV-51 [8/35 (22.8%)], HR-HPV types 18 and 39 [7/35 (20.0%)] and LR-HPV-43 [6/35 (17.1%)]. HR-HPV-52 and LR-HPV-44 were detected in lower proportions [5/35 (14.3%) and 4/35 (11.4%), respectively]. LR-HPV-42, LR-HPV-54, HR-HPV-31 and HR-HPV-35 were detected in very low proportions [3/35 (8.5%)]. Multiple HR-HPV infections were diagnosed in one-third of anal samples [16/50 (32.0%)], including around half of HR-HPV-positive anal swabs [16/35 (45.7%)]. More than half [27/50 (54.0%)] swabs were infected by at least one of HPV genotypes targeted by Gardasil-9® vaccine, including a majority of vaccine HR-HPV [22/50 (44.0%)]. In multivariate analysis, participation to sex in group was associated with anal infection by multiple HPV (aOR: 4.5, 95% CI: 1.1–18.1%; *P* = 0.032), and HIV-1 infection was associated with anal shedding of multiple HR-HPV (aOR: 5.5, 95% CI: 1.3–24.5%; *P* = 0.024).

**Conclusions:**

These observations indicate that the MSM community living in Bamako is at high-risk for HR-HPV anal infections, with a unique epidemiological HPV genotypes profile and high prevalence of anal HPV covered by the Gardasil-9® vaccine. Scaling up prevention strategies against HPV infection and related cancers adapted to this highly vulnerable MSM community should be urgently prioritized with innovative interventions.

## Introduction

The human papillomavirus (HPV) is responsible for a significant disease burden in men who have sex with men (MSM), including benign and malignant lesions [1]. Anogenital warts, cancers of the penis, anus and oropharynx represent a relevant health problem in MSM populations worldwide [1]. Overall, oncogenic high-risk (HR)-HPV genotypes are responsible for the majority of anal cancers [2, 3].

In sub-Saharan Africa, the MSM populations have been identified as core groups for several sexually transmitted infections (STIs), including human immunodeficiency virus (HIV) [4, 5], herpes simplex virus 2, and HPV [6, 7]. The burden of HPV infection among MSM living in sub-Saharan Africa, although nonetheless poorly documented, is important [7]. Thus, the reported prevalences of anal HR-HPV infection among MSM living in sub-Saharan Africa appear to be higher than those usually recorded in studies conducted in developed countries, which range from 20.9% to 65% [8, 9]. Recent reports from South Africa [10] and Nigeria [11] highlight very high prevalence of anal HR-HPV infection, ranging from 57.6% to 70.1%, among MSM living in sub-Saharan Africa, particularly among those co-infected with HIV [10, 11]. Anal HR-HPV infection in African MSM was strongly associated with high-risk sexual behaviors such as having sex with men only, participating to group sex, and having receptive anal sex without a condom [10, 11]. Interestingly, a great diversity of predominant HPV genotypes has been observed in South African and Nigerian studies, suggesting the possibility of unique spatial distributions of HPV diversity by region in sub-Saharan Africa [10, 11]. Müller and colleagues described in South Africa a distribution quite similar to that commonly observed in the world with HPV-16 as the predominant genotype [10]. In contrast, Nowak and colleagues depicted an atypical distribution profile in Nigeria with non-vaccine HR-HPV-35 as the predominant genotype circulating in MSM [11]. Although limited, these observations highlight that MSM in sub-Saharan Africa constitute a central group at high-risk for HPV infections and that the distribution of the main HPV genotypes involved in anal cancers in African MSM may be quite different from that generally observed. Finally, in order to implement effective prevention based on prophylactic HPV vaccine adapted to each region of sub-Saharan Africa, it is important to establish the molecular distribution of the predominant genotypes of HR-HPV circulating in African MSM.

High prevalences of anal HR-HPV infections in MSM living in West Africa, including Burkina Faso [12], Liberia [13], Mali [12], Nigeria [11, 14], Togo [15], and Senegal [16], have been reported. Since the epidemiological situation of HPV infection in MSM may be specific in a given area [7], with implication on prophylactic HPV vaccine efficacy, we herein designed a cross-sectional study to assess the prevalence and genotypes distribution of anal HPV and associated risk factors, including sexual behavior, in a population of HIV-infected and non-HIV-infected MSM living in Bamako, the capital of Mali.

## Material and Methods

### Study Population, Medical Interventions, and Data Collection

The Clinic of the National NGO Soutoura, Bamako, Mali is a specialized care center exclusively dedicated to key populations. It offers counseling, testing, care, and support to MSM from Bamako. MSM regularly attend the center for HIV and STI screening and care, to receive specific treatment, HIV counseling and HIV global support for those tested positive. For purposes of the study, a specific strategy involving peer educators was adopted in order to confirm the accuracy of homosexuality of the included MSM. Thus, inclusion criteria were to be in majority age (age ≥ 18 years), to be approved as having sex with men by his peers, to get possible follow up for at least 3 months, to have a fully informed medical and socio-demographic record, and to sign the informed consent form. Refusal to participate and age under 18 were set as exclusion criteria.

At inclusion, a standardized interview was conducted to collect socio-demographic characteristics and behavioral data, including age, number of male sexual partners in the past 6 months, sexual orientation, frequency of condom use, sexual practices, HIV status and antiretroviral treatment for those who were already aware of their positive HIV status and finally to advise participants on HIV and associated STIs.

After the interviews, MSM undertook medical appointments including clinical examinations and biological investigations for the diagnosis of the most common STIs including HIV (for those who did not know their HIV-serological status), syphilis and hepatitis B. Biological results were returned 72 hours after and those positive for STIs received adapted treatment. HIV-positive MSM were enrolled in the HIV cohort followed in the center. The medical intervention package consisted of HIV/STI counseling, condom distribution, clinical examination, biological monitoring, and medical care for patients infected with STIs and HIV. A medical professional carried out physical and clinical examinations to check patients for symptoms of potential diseases. For HIV/STI counseling and condom distribution, a 7 to 10-minute interactive conversation on HIV, STIs, their modes of transmission and effective prevention mean with a special emphasis on condom use as an easy and effective prevention tool, was carried out by health care assistants. At the end, the health care assistants distributed as many condoms as required to the participants.

### Samples and Processing

Plasma or serum samples from blood collected by venipuncture in each MSM have been used for serological testing for HIV infection, as recommended by the national algorithm of the Mali HIV National Control Program, using in series OnSite HIV1/2 Ab Plus Combo Rapid Test (CTK Biotech Inc., Poway, Ca, USA) for HIV screening and ImmunoComb® II HIV 1&2 BiSpot (Orgenics Ltd., Yavne, Israël) for confirmation [17]. Samples for HPV molecular testing and genotyping were obtained by inserting a wet cotton swab into the anal canal, rotating 5 times and then removing. The swab was immediately placed in a sample tube then put in a cooler containing frozen ice packs, and finally frozen at − 80 °C within 1 hour. Frozen swabs were further send in ice in the Laboratoire de Virologie, hôspital Européen Georges Pompidou, Paris, France, for HPV detection and genotyping. All samples were kept frozen at − 80 °C before the DNA extraction procedure.

### HPV Detection and Genotyping

DNA was extracted from the anal swab sample using the DNeasyBlood and Tissue kit, as recommended by the manufacturer (Qiagen, Hilden, Germany). HPV DNA detection and genotype distribution were performed using the Anyplex™ II HPV28 detection assay (Seegene, Seoul, South Korea), as previously described [18]. The Anyplex™ II HPV28 detection test distinguishes 28 genotypes of HPV, including 13 high-risk types (HR-HPV -16, -18, -31, -33, -35, -39, -45, -51, -52, -56, -58, -59 and -68), 9 low-risk types (LR) (LR-HPV -6, -11, -40, -42, -43, -44, -53, -54 and -70), then 6 genotypes reported as possibly carcinogenic (HPV-26, -61, -66, -69, -73 and -82).

### Statistical Analyzes

Statistical analyzes were performed using SAS 9.4 (SAS Institute Inc., NC, USA). *P* values ​​were calculated using Fisher’s exact test for categorical variables and using Mann-Whitney *U*-test for quantitative variables. Logistic regression models were assessed to evaluate the association of each independent variable [*i.e.*, age at enrollment, HIV-1 infection, sex of sexual partner (MSM-exclusively or MSMW), the number of sexual male partners in the last 6 months, and sexual practices in the last 6 months (condomless receptive anal sex; condomless insertive anal sex; regular receptive oral sex)] with the HPV type-specific anal infections (*i.e.*, anal infection by any type of HPV, multiple types of HPV, HR-HPV and multiple HR-HPV). All variables statistically significant (*P* < 0.05) in univariate analyses were entered into multivariate logistic regression models. Crude Odds ratio (cOR) and adjusted Odds ratio (aOR) were calculated, as appropriate along with 95% confidence intervals (CI). For variable giving infinite OR, the Odds ratios and their confidence intervals were recalculated, using the statistical software package R (available at https://www.r-project.org/) and the hypothesis test inversion method, as previously described [19]. The final multivariate model for any HPV outcome included condomless receptive and insertive anal sex and regular receptive oral sex. For multiple HPV outcome, the final multivariate model included condomless receptive and insertive anal sex and regular receptive oral sex. For HR-HPV outcomes, the final multivariate model included condomless insertive anal sex and regular receptive oral sex. Finally, for the multiple HR-HPV outcomes variable, the final multivariate model included HIV infection and condomless insertive anal sex.

### Ethics Statement

The approval of the study was obtained from the Scientific Committee of the University of Bamako, Mali, which constitutes the national Ethics Committee. All participating MSMs were of legal age and their informed consent was obtained before each questionnaire was documented.

## Results

### Characteristics of Study Population

Fifty participants were included from April to September 2019 and their socio-demographic, sexual, clinical and biological characteristics are presented in Table 1. Among them, 16 (32.0%) were infected with HIV-1 and 34 (68.0%) were HIV-negative. The majority of HIV-positive participants were yet followed up at Clinic of the National NGO Soutoura (90.0%) and took highly active antiretroviral therapy (87.5%).

**Table 1:** Baseline characteristics according to sexual behavior and HIV serostatus among the 50 study men who have sex with men (MSM) living in Bamako, Mali

Characteristics	Overall	MSM-exclusively	MSMW	P*	HIV-	HIV+	P*
Number	50	38	12		34	16	
Age [mean (SD), years] < 20 20–29 ≥ 30	24 (4.8) 12 (24.0) 30 (60.0) 8 (16.0)	24 (4.7) 10 (26.3) 23 (60.5) 5 (13.2)	26 (5.2) 2 (16.7) 7 (58.3) 3 (25.0)	*0.244*	23 (4.5) 11 (32.4) 20 (58.8) 3 (8.8)	27 (5.0) 1 (6.2) 10 (62.5) 5 (31.3)	*0.018*
Age at first intercourse [mean (SD), years] < 15 15–17 ≥ 18	16 (2.2) 7 (14.0) 24 (48.0) 19 (38.0)	16 (2.2) 6 (15.8) 18 (47.4) 14 (36.8)	17 (2.2) 1 (8.3) 6 (50.0) 5 (41.7)	*0.200*	17 (2.2) 4 (11.8) 17 (50.0) 13 (38.2)	16 (2.3) 3 (18.7) 7 (43.8) 6 (37.3)	*0.519*
HIV infection [n (%)] CD4 T cell count [median (range), cells/μL] Under HAART [n (%)]	16 (32.0) 570 (327–978) 14 (87.5)	12 (31.6) 541 (327–842) 10 (83.3)	4 (33.3) 645 (469–978) 4 (100.0)	*0.995*	NA	NA	
Family situation [n (%)] Single Couple	38 (76.0) 12 (24.0)	NA	NA	NA	26 (76.5) 8 (23.5)	12 (75.0) 4 (25.0)	*0.999*
Number of sexual partners in last 6 months [n (%)] 1–5 > 5	40 (80.0) 10 (20.0)	28 (73.7) 10 (26.3)	12 (100.0) 0 (0.0)	*0.092*	27 (79.4) 7 (20.6)	13 (81.3) 3 (18.7)	*1.0*
Use of condom [n (%)] Never Sometimes Always	3 (6.0) 46 (82.0) 1 (2.0)	1 (2.5) 36 (94.5) 1 (2.5)	2 (16.7) 10 (83.3) 0 (0.0)	*0.768*	2 (5.9) 31 (91.2) 1 (2.9)	1 (6.2) 15 (93.8) 0 (0.0)	*0.876*
Engaged in group sex [n (%)]	18 (36.0)	15 (39.5)	3 (25.0)	*0.497*	11 (32.4)	7 (43.7)	*0.532*
Practicing ChemSex [n (%)]	12 (24.0)	12 (31.6)	0 (0.0)	*0.046*	8 (23.5)	4 (25.0)	*1.0*
Receptive anal intercourse [n (%)]	40 (80.0)	31 (81.6)	9 (75.0)	*0.685*	25 (73.5)	15 (93.8)	*0.094*
Insertive anal intercourse [n (%)]	34 (68.0)	25 (65.8)	9 (75.0)	*0.727*	21 (61.8)	13 (81.3)	*0.208*
Receptive oral sex [n (%)]	36 (72.0)	26 (68.4)	10 (83.3)	*0.468*	22 (64.7)	14 (87.5)	*0.175*
Insertive oral sex [n (%)]	43 (86.0)	33 (86.8)	10 (83.3)	*0.789*	27 (79.4)	16 (100.0)	*0.081*
STI gonorrhea [n (%)]	20 (40.0)	17 (44.7)	3 (25.0)	*0.316*	12 (35.3)	8 (50.0)	*0.366*
STI *Chlamydia* [n (%)]	7 (14.0)	7 (18.4)	0 (0.0)	*0.174*	4 (11.8)	3 (18.7)	*0.665*

^*^ *P*-value calculated using Pearson’s χ2 test or Fisher’s exact test for categorical variables and the non-parametric Mann-Whitney *U*-test for non-categorical variables

*HAART* Highly active antiretroviral therapy, *MSM* men who have sex with men, *MSM-exclusively* men who have sex only with men, *MSMW* men who have sex with both men and women, *NA* Not applicable, *NS* Not significant

Study MSM were exclusively Malian, and mainly young men (mean age, 24 years; range, 18–35). HIV-infected MSM were older than HIV-negative MSM. The average age of participants for the first sexual intercourse was 16-year-old. The majority of participants [38/50 (76%)] reported being single, while a minority [12/50 (24%)] were in a relationship. Most [40/50 (80%)] of MSM reported having had sex with at least 1 to 5 partners in the past 6 months and most of them [46/50 (82%)] used only sometimes condoms during sex. The prevalence of STIs was high in study population. Sexual practices were not significantly different between exclusive MSM and men having sex with both men and women (MSMW). HIV-infected MSM reported practicing receptive oral sex more frequently than HIV-negative MSM [14/16 (87.5%) *versus* 22/34 (64.7%)], however without statistical differences.

### HPV Prevalence and Genotypes Distribution

**Fig. 1:**
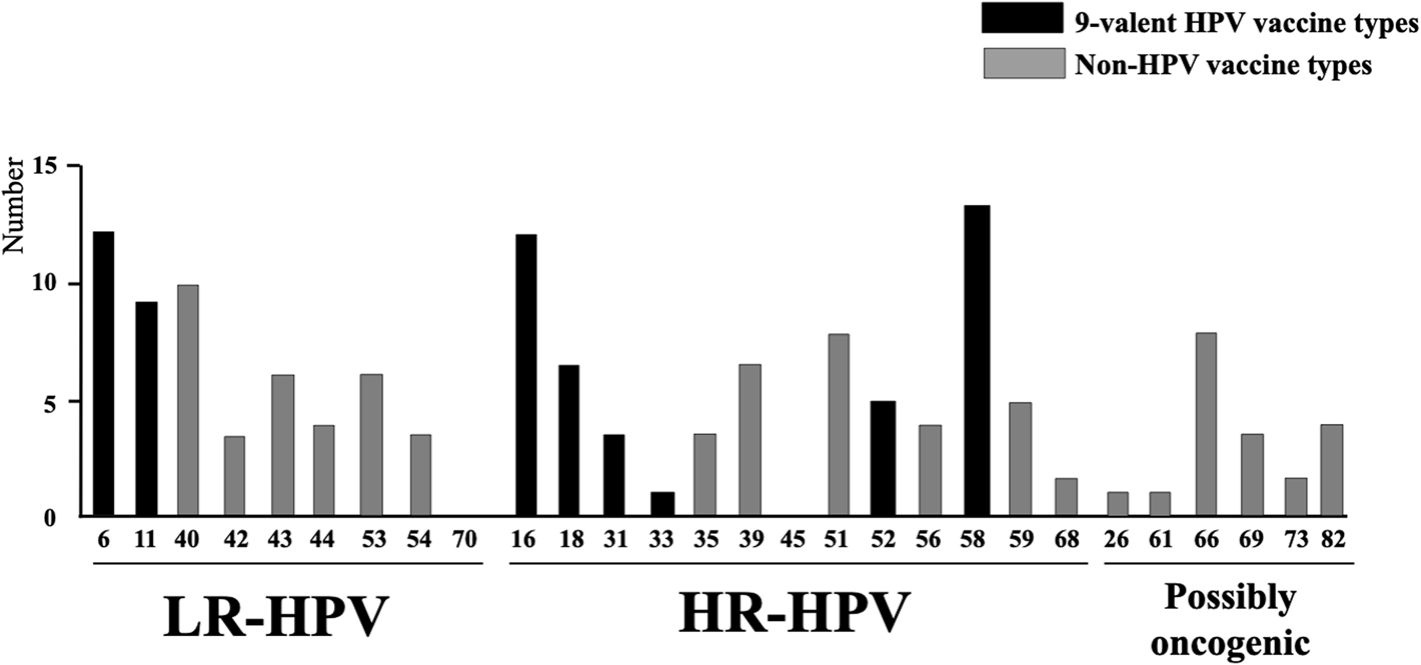
Distribution of the types of anal HPV included (or not included) in the Gardasil-9 vaccine. Number of high-risk (HR) and low-risk (LR) HPV genotypes in 35 anal swabs positive for HPV DNA by molecular biology based on their possible prevention by the 9-valent HPV vaccine study among men who have sex with men (*n* = 50) living in Mali. *Nota bene.* The 9-valent Gardasil® vaccine (Merck & Co. Inc., New Jersey, USA) is effective against HPV genotypes 6, 11, 16, 18, 31, 33, 45, 52 and 58

As shown in Table 2 and Fig. 1, the overall prevalence of anal HPV infection of any genotypes in the study population was 70.0% (35/50) with 80.0% (28/35) of samples positive for HR-HPV DNA. Most [28/50 (56.0%)] of the anal swabs contained multiple HPV genotypes and 57.1% (16/28) of them contained an average of 3.7 HR-HPV per anal swab specimen. The majority [28/35 (80.0%)] of HPV-positive samples showed at least 1 HR-HPV.

**Table 2:** HPV test results according to sexual behavior and HIV serostatus among the 50 study men who have sex with men (MSM) living in Bamako, Mali

Characteristics	Overall	MSM-exclusively	MSMW	P*	HIV-	HIV+	P*
Number	50	38	12		34	16	
Any anal HPV [n (%)]	35 (70.0)	25 (65.8)	10 (83.3)	*0.304*	21 (61.8)	14 (87.5)	*0.098*
Multiple anal HPV [n (%)]	28 (56.0)	21 (55.3)	7 (58.3)	*1.0*	15 (44.1)	13 (81.3)	*0.016*
Anal HR-HPV [n (%)]	28 (56.0)	21 (55.3)	7 (58.3)	*1.0*	16 (47.1)	12 (75.0)	*0.076*
Multiple HR-HPV [n (%)]	16 (32.0)	12 (31.6)	4 (33.3)	*1.0*	7 (20.6)	9 (56.3)	*0.021*
HPV-16 [n (%)]	12 (24.0)	6 (15.7)	6 (50.0)	*0.704*	5 (14.7)	7 (43.7)	*0.036*
HPV-18 [n (%)]	7 (14.0)	4 (10.5)	3 (25.0)	*0.336*	4 (11.8)	3 (18.7)	*0.665*
HPV-16 and HPV-18 [n (%)]	4 (8.0)	2 (5.2)	2 (16.7)	*0.239*	2 (5.9)	2 (12.5)	*0.584*
Any 4-valent vaccine HPV [n (%)]^μ^	24 (48.0)	17 (44.7)	7 (58.3)	*1.0*	14 (41.2)	10 (62.5)	*0.574*
Multiple 4-valent vaccine HPV [n (%)]	12 (24.0)	8 (21.0)	4 (33.3)	*0.876*	6 (17.6)	6 (37.5)	*0.658*
Any 9-valent vaccine HPV [n (%)]^£^	27 (54.0)	18 (47.4)	9 (75.0)	*0.321*	16 (47.0)	11 (68.7)	*0.256*
Multiple 9-valent vaccine HPV [n (%)]	21 (42.0)	14 (36.8)	7 (58.3)	*0.379*	12 (35.3)	9 (56.2)	*0.301*
Any 4-valent vaccine HR-HPV [n (%)]^μ^	15 (30.0)	12 (31.6)	3 (25.0)	*0.899*	8 (23.5)	7 (43.7)	*0.125*
Multiple 4-valent vaccine HR-HPV [n (%)]	8 (16.0)	6 (15.8)	2 (16.7)	*1.0*	5 (14.7)	3 (18.7)	*0.573*
Any 9-valent vaccine HR-HPV [n (%)]^£^	22 (44.0)	15 (39.4)	7 (58.3)	*0.954*	13 (38.2)	9 (56.2)	*0.206*
Multiple 9-valent vaccine HR-HPV [n (%)]	16 (32.0)	12 (31.6)	4 (33.3)	*1.0*	10 (29.4)	6 (37.5)	*0.678*

^*^ *P*-value calculated using Pearson’s χ2 test or Fisher’s exact test for categorical variables and the non-parametric Mann-Whitney *U*-test for non-categorical variables

^μ^ The 4-valent Gardasil-4® vaccine (Merck & Co. Inc., New Jersey, USA) is effective against HPV genotypes 6, 11, 16 and 18

^£^ The 9-valent Gardasil-9® vaccine is effective against HPV genotypes 6, 11, 16, 18, 31, 33, 45, 52 and 58

*HR-HPV* high-risk human papillomavirus, *LR-HPV* low risk human papillomavirus, *MSM* men who have sex with men, *MSM-exclusively* men who have sex only with men, *MSMW* men who have sex with both men and women, *NS* Not significant

HR-HPV-58 was the most detected genotype [13/35 (37.1%)], followed by HR-HPV-16 and LR-HPV-6 [12/35 (34.2%)], LR-HPV-40 [10/35 (28.6%)], LR-HPV-11 [9/35 (25.7%)], HR-HPV-51 [8/35 (22.8%)], HR-HPV types 18 and 39 [7/35 (20.0%)] and LR-HPV-43 [6/35 (17.1%)]. HR-HPV-52 and LR-HPV-44 were detected in lower proportions [5/35 (14.3%) and 4/35 (11.4%), respectively]. LR-HPV-42, LR-HPV-54, HR-HPV-31 and HR-HPV-35 were detected in very low proportions [3/35 (8.5%)] (Fig. 1). Multiple HR-HPV infections were diagnosed in one-third of anal samples [16/50 (32.0%)], including around half of HR-HPV-positive anal swabs [16/35 (45.7%)]. HPV-16 and/or HPV-18 were detected in around one-third [15/50 (30.0%)] of anal samples, including a minority [4/50 (8.0%)] infected with both HPV-16 and HPV-18. HPV-16 and HPV-18 were found in less than half [15/35 (42.8%)] anal samples positive for HR-HPV.

Of the 14 HIV-1-infected MSM with anal HPV, the quasi-totality [13/14 81.3%)] had multiple HPV infection and 2 (16.7%) were coinfected with HR-HPV types 16 and 18 (Table 2). HIV-infected MSM showed significantly more frequent multiple anal HPV, multiple anal HR-HPV genotypes and HPV-16 infection than HIV-negative MSM (multiple HPV: 81.3% *versus* 44.1%, *P* = 0.0168; multiple HR-HPV: 56.3% *versus* 20.6%, *P* = 0.0214; HPV-16: 43.7% *versus* 14.7%, *P* = 0.0363). MSM-exclusively were less infected with anal HPV [25/38 (65.8%)] than bisexual MSM [10/12 (83.3%)], but the difference was not significant.

Possible efficiencies of anal HPV prevention by 4- and 9- valent Gardasil® vaccines were further assessed (Table 2 and Fig. 1). Around half [24/50 (48.0%)] anal samples were infected by at least one HPV genotypes targeted by the Gardasil-4® vaccine, and around one-third [15/50 (30.0%)] of HPV-positive anal samples harbored at least 1 HR-HPV 4-valent Gardasil® genotype. Anal HPV genotypes targeted by the 9-valent prophylactic Gardasil-9® vaccine were more frequent than those targeted by Gardasil-4® vaccine, with a little more than half [27/50 (54.0%)] anal samples, including a majority of vaccine HR-HPV [22/50 (44.0%)]. Among 9-valent Gardasil® vaccines genotypes, HPV-58 was the most detected in the study population [13/35 (37.1%)], followed by HPV-16 [12/35 (34.3%)], -18 [7/35 (20.0%)], -52 [5/35 (14.2%)], -31 [3/35 (8.6%)] and -33 [1/35 (2.8%)] (Fig. 1). More than half (53.6%; 15/28) of anal HR-HPV infections would be prevented by Gardasil-4® vaccine and more than three-quarters (78.6%; 22/28) by the Gardasil-9® vaccine.

**Fig. 2:**
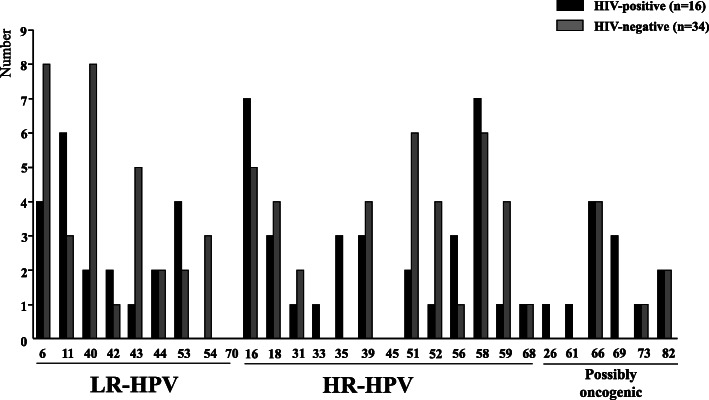
Distribution of anal HPV types according to the HIV-serostatus. Number of high-risk (HR) and low-risk (LR) HPV genotypes in anal swab positive for HPV DNA by molecular biology according to HIV-serostatus among study men who have sex with men (n = 50) living in Bamako, Mali

HIV-infected MSM were found to be significantly more infected with HPV and HR-HPV genotypes targeted by 4- and 9- valent Gardasil® vaccines than HIV-negative MSM, although the differences did not reach significance (Table 2 and Fig. 2).

### Risk Factors Associated with Anal HPV Shedding

The associations between anal HPV infection, including anal infection by any type of HPV, multiple types of HPV, HR-HPV type and multiple types HR-HPV, with their potential risk factors were assessed by logistic regression analysis, as shown in Table 3.

**Table 3:** Univariate and multivariate logistic regression analyses for HPV-associated risk factors 50 study men who have sex with men (MSM) living in Bamako, Mali

	Any HPV					Multiple HPV					HR-HPV					Multiple HR-HPV				
Number of patients	35					28					28					16				
Risk factors	n (%)	cOR (95%CI)	P*	aOR (95%CI)	P*	n (%)	cOR (95%CI)	P*	aOR (95%CI)	P*	n (%)	cOR (95%CI)	P*	aOR (95%CI)	P*	n (%)	cOR (95%CI)	P*	aOR (95%CI)	P*
Age (years)																				
18–19	7 (20.0)	1	0.734	NA	NA	5 (17.9)	1	0.277	NA	NA	5 (17.9)	1	0.838	NA	NA	2 (12.5)	1	0.192	NA	NA
20–29	23 (65.7)	2.4 (0.6–8.6)				19 (67.8)	2.4 (0.7–8.5)				19 (67.8)	2.4 (0.7–8.5)				12 (75.0)	3.3 (0.7–16.6)			
≥ 30	5 (14.3)	1.2 (0.3–4.9)				4 (14.3)	1.4 (0.4–5.7)				4 (14.3)	1.4 (0.4–5.7)				2 (12.5)	1.7 (0.3–9.9)			
HIV-1 infection	14 (40.0)	4.3 (1.2–15.2)	0.022	3.1 (0.6–17.3)	0.188	13 (46.4)	5.5 (1.8–16.8)	0.002	5.5 (1.3–24.5)	0.024	12 (42.9)	3.4 (1.2–9.6)	0.022	3.2 (0.8–12.3)	0.090	9 (56.3)	5.0 (1.7–14.7)	0.003	5.5 (1.4–21.4)	0.014
Family situation																				
Single	25 (71.4)	1	0.138	NA	NA	21 (75.0)	1	0.812	NA	NA	21 (75.0)	1	0.812	NA	NA	12 (75.0)	1	0.885	NA	NA
Couple	10 (28.6)	2.6 (0.7–9.2)				7 (25.0)	1.3 (0.4–3.2)				7 (25.0)	1.1 (0.4–3.2)				4 (25.0)	1.1 (0.4–3.2)			
Number of sexual partners in last 6 months																				
1–5	28 (80.0)	1	1.0	NA	NA	23 (82.1)	1	0.582	NA	NA	23 (82.1)	1	0.582	NA	NA	15 (93.8)	1	0.058	1	0.110
> 5	7 (20.0)	1.0 (0.3–3.2)				5 (17.9)	0.9 (0.7–1.9)				5 (17.9)	0.9 (0.7–1.9)				1 (6.2)	1.1 (0.2–1.5)		1.1 (0.2–1.5)	
Engaged in group sex	15 (42.9)	3.0 (1.0–9.3)	0.057	NA	NA	14 (50.0)	4.5 (1.6–12.9)	0.005	4.5 (1.1–18.1)	0.032	13 (46.4)	3.0 (1.1–8.1)	0.035	2.8 (0.8–10.0)	0.115	6 (37.5)	1.1 (0.4–3.1)	0.854	NA	NA
Receptive anal intercourse	31 (88.6)	5.2 (1.3–20.3)	0.018	3.1 (0.6–15.9)	0.178	25 (89.3)	3.9 (0.9–16.2)	0.062	NA	NA	24 (85.7)	2.3 (0.6–8.6)	0.236	NA	NA	15 (93.8)	5.4 (0.7–44.8)	0.118	NA	NA
Insertive anal intercourse	25 (71.4)	1.7 (0.5–5.3)	0.382	NA	NA	23 (82.1)	2.1 (0.7–6.3)	0.193	NA	NA	21 (75.0)	2.1 (0.7–6.7)	0.195	NA	NA	10 (62.5)	0.7 (0.2–2.2)	0.530	NA	NA
Receptive oral sex	28 (80.0)	3.5 (1.1–11.5)	0.038	1.9 (0.4–8.4)	0.417	23 (82.1)	3.2 (1.0–10.5)	0.057	NA	NA	22 (78.6)	2.1 (0.7–6.7)	0.211	NA	NA	14 (87.5)	3.8 (0.8–18.4)	0.094	NA	NA
Insertive oral sex	29 (82.9)	0.4 (0.1–3.0)	0.335	NA	NA	25 (89.3)	1.9 (0.4–8.8)	0.438	NA	NA	25 (89.3)	1.9 (0.4–8.8)	0.438	NA	NA	14 (87.5)	1.2 (0.2–6.6)	0.828	NA	NA
STI gonorrhea or *Chlamydia*	16 (45.7)	2.3 (0.8–6.8)	0.125	NA	NA	13 (46.4)	1.9 (0.7–4.9)	0.209	NA	NA	12 (42.9)	1.31 (0.5–3.4)	0.577	NA	NA	8 (50.0)	1.8 (0.7–5.1)	0.247	NA	NA

* *P*-value calculated using Pearson’s χ^2^ test or Fisher’s exact test for categorical variables and the non-parametric Mann-Whitney U-test for non-categorical variables

** NA: Not attributable for variables giving crude Odds ratio not significant in univariate analysis (*P* > 0.05)

*aOR* adjusted Odds ratio, *cOR* crude Odds ratio, *HIV-1* Human immunodeficiency virus-1, *HR-HPV* high-risk human papillomavirus, *LR-HPV* low-risk human papillomavirus, *MSM* men who have sex with men, *n* Number (size of study group), *CI* Confidence Interval

In the univariate analysis, anal infection by any HPV was significantly associated with the practice of condomless receptive anal sex (cOR: 5.2, 95% CI: 1.3–20.3%; *P* = 0.018) and receptive oral sex (cOR: 3.5, 95% CI: 1.1–11.5%; *P* = 0.038) and HIV-infection (cOR: 4.3, 95% CI: 1.2–15.2%; *P* = 0.022); anal infection with multiple HPV as well as anal infection with HR-HPV genotypes were both associated with HIV infection (cOR: 5.5, 95%CI: 1.8–16.8%; *P* = 0.002 and cOR: 3.4, 95% CI: 1.2–9.6%; P = 0.022, respectively) and the practice of group sex (cOR: 4.5, 95% CI: 1.6–12.9%; *P* = 0.005 and cOR:3.0, 95% CI: 1.1–8.1%; *P* = 0.035, respectively); finally anal infection with multiple HR-HPV was associated with HIV infection (cOR: 5.0, 95% CI: 1.7–14.7%; *P* = 0.003).

In the multivariate analysis, only being infected with HIV and having participated in group sex party remained significantly linked to anal infection with multiple HPV infection (aOR: 5.5, 95% CI: 1.3–24.5%; *P* = 0.024 and aOR: 4.5, 95% CI: 1.1–18.1%; *P* = 0.032).

## Discussion

The present study assessing the epidemiological features of anal HPV infection within the MSM community living in Mali, followed up at the main care center exclusively dedicated to HIV key populations in Bamako, the Clinic of the National NGO Soutoura, showed remarkable findings. Firstly, the prevalence of anal HPV was also particularly high (70.0%) and unique due to the high prevalence of HR-HPV (80.0%), high genotypes diversity and frequent (45.7%) multiple HR-HPV infections among HPV-positive samples. Secondly, the distribution of anal HPV appeared specific to study population, the most prevalent genotypes being HR-HPV-58, followed by HPV-6, HPV-16, HPV-40, HPV-11, HPV-51, HPV-18 and HPV-39, while HPV-43, HPV-52 and HPV-44 were in lower proportions, and HPV-42, HPV-54, HPV-31 and HPV-35 in very low proportion. The classical HR-HPV-16 and HR-HPV-18 were only present in less than half (42.8%) of HR-HPV-positive anal samples, likely indicating possible regional clusterization in the diffusion of certain HR-HPV genotypes within the MSM community living in Bamako. Thirdly, all HPV types targeted by the prophylactic Gardasil-9® vaccine, except HPV-45, were detected in the majority (78.6%) of HR-HPV-positive anal samples, suggesting that the current 9-valent vaccine could be beneficial for the prevention of HPV-associated disease in the majority of MSM community living in Bamako, although around one-fifth of HPV anal infection would not be prevented. Finally, the practice of sex group was significantly associated with infection by multiple anal HPV, and HIV infection with the anal carriage of HR-HPV. Taken together, our observations indicate for the first time that the MSM community living in Bamako should be at very high-risk for HR-HPV anal infections, and strongly suggest that scaling up prevention strategies against HPV infection and related cancers adapted to this highly vulnerable MSM community should be urgently prioritized with innovative interventions.

The prevalence of HIV-1 infection in study MSM was notably high (32.0%) compared to the national HIV prevalence in Mali, which is 1.1% [20]. HIV prevalence among MSM in Bamako was estimated in 2015 at 13.7% using respondent driven sampling on a large cohort of 552 MSM, eight times the prevalence among men in general population from the same city [21, 22]. The HIV prevalence found in our study was more than twice higher, probably linked to a recruitment bias, since the Clinic of the National NGO Soutoura preferentially deals with key populations in relation to the HIV risk.

The prevalence of anal HPV was particularly high in this sample of MSM from the Bamako’s community. The high prevalence of anal HR-HPV in our study MSM appeared quite similar to the prevalence reported in MSM living in Liberia, Togo, South Africa, Central African Republic and Nigeria, ranging from 45% to 72% [10–13, 15, 18], but lower than the prevalence reported in young Black American MSM (87%) [23]. Other reports conducted outside Africa showed relatively high anal HR-HPV prevalence rates, ranging from 29% to 56% [24–27]. Overall, anal HR-HPV infection was up to 4–10 times more frequent in MSM living in many countries outside Africa than in heterosexual men [28, 29]. Finally, our observations confirm that anal HPV constitutes a major infectious health concern in the MSM living in Bamako, highly escalated by HIV infection, and that each MSM community is characterized by local epidemiological specificities rendering necessary their epidemiological characterization before the implementation of any intervention.

In our series, the distribution of anal HPV in anal samples appeared specific and unique, with a mixture of HR-HPV and LR-HPV. Among HR-HPV, HPV-58 (37.1%) was the predominant genotype, followed by HPV-16 with a very close prevalence (34.2%), and HPV -51 (22.8%), HPV − 18 and − 39 (20.0%). This distribution partly mirrors the previous observations by Nowak and colleagues, reporting that anal samples from MSM living in Nigeria harbored HPV-58 as the most prevalent 9-valent vaccine genotype followed by HPV-16 and HPV-18 as minor genotypes [11]. Similarly, in the Central African Republic, Mboumba Bouassa and colleagues reported in MSM living in Bangui that the most prevalent 9-valent vaccine HR-HPV was HPV-58, while HPV-16 and HPV-18 were very poorly represented [18]. Study MSM in Bamako harbored however a very low prevalence of HPV-35 (8.5%), which was in contrast highly represented in the Central African Republic [18] and Nigeria [11, 14]. Finally, the anal HR-HPV distribution in study MSM living in Bamako appeared intermediate between the most frequent genotypes distribution usually observed in Western countries and South Africa, and some unique distribution of rare genotypes reported in some sub-Saharan African countries. Thus, HR-HPV distribution in our MSM series showed relatively high prevalence of HR-HPV-58 as previously reported in sub-Saharan Africa [11, 14, 18] in association with relatively high frequency of HPV-16 as previously reported in MSM living in South Africa [10], and with low frequency of poorly encountered genotypes such as HPV-35 which is thought to be clusterized in certain African MSM population in sub-Saharan Africa [11, 14, 18]. Taken together, these observations suggest the possibility of a regional distribution in molecular epidemiology of HR-HPV within the diverse MSM communities inside the sub-Saharan African continent [7]. Thus, it is possible to hypothesize that anal cancers in certain black African MSM populations may be due to other HR-HPV rather than HPV-16 and HPV-18, which constitute the most frequently HR-HPV types involved in anal cancers in MSM living in Western countries [30, 31]. In Mali, there is no data on the HPV type specific prevalence in the general population. It would be interesting to check whether the particular HPV genotype distribution found in our Malian MSM series is similar to that in the female or general population. Further studies are nevertheless needed to determine the natural history and the burden of HPV-associated diseases in black African MSM in order to confirm our observations and to formulate effective and adapted HPV vaccine strategies towards young African MSM.

The coverage offered by the Gardasil-9® vaccine in study MSM was much higher than that given by the Gardasil-4® vaccine. Thus, HPV types targeted by the prophylactic Gardasil-9® vaccine were detected in more than half (54.0%) of HPV-positive anal samples. All types of HPV targeted by the Gardasil-9® vaccine were detected in the majority (78.6%) of HR-HPV-positive anal samples, except the HPV-45 which was not detected, and 42% of HPV-positive anal specimens contained multiple 9-valent HPV vaccine types. Similarly, high rates of 9-valent HPV vaccine types in anal canal of MSM were previously reported in South Africa (57%) [10] and Central African Republic (68.9%) [18]. These observations indicate that MSM living in Bamako, as other MSM populations, constitute a key target population for HPV vaccination with the current prophylactic Gardasil-9® vaccine, which would potentially prevent most of HPV infections and associated anal diseases. However, anal HR-HPV not included in the prophylactic 9-valent vaccine, including HPV-39, HPV-51 and HPV-35, were found in one-fifth (21.4%) of study MSM, indicating that the current HPV vaccine may be insufficient to prevent HPV-related diseases in a significant proportion of the MSM community living in Mali. Thus, the guidelines on HPV immunization recommended in 2015 by the American Cancer Society (ACS), which integrate HPV vaccination up to 26 years for young MSM with the current two large spectrum HPV vaccines [32, 33], because HPV-16 and HPV-18 are mostly involved in HPV-associated anal cancer in Western countries [30, 31], may be only partially adapted to the MSM community living in Mali and other sub-Saharan African settings.

Among various evaluated risk factors, participation to sex group was associated with anal infection by multiple HPV, and HIV infection was associated with anal shedding of multiple HR-HPV, after adjusting for confounders and other variables found significant in the univariate analysis. The other possible associations did not reach statistical differences. Thus, the population of study HIV-positive MSM living in Bamako constituted a high-risk group accumulating several risky sexual behaviors and multiple anal HR-HPV infections.

Sex group was associated with higher risk of anal HPV shedding in study MSM. This finding is reminiscent with the increased sexual disinhibition among MSM community in Cape Town, South Africa, in whom being engaged in group sex in lifetime was associated with 4.7-fold-risk having any anal HPV infection and 3.1-fold-risk of having a greater likelihood of anal infection by a HR-HPV type [10]. Furthermore, study MSM only used condoms inconsistently. These findings confirm that the risk of anal HPV acquisition is strongly associated with high-risk sexual behavior in the MSM population. Indeed, MSM practicing condomless anal intercourse with male partners are more likely to acquire HIV, various STIs and anal HPV than those who only practice condomless insertive anal intercourse, with exacerbation in direct relationship to the number of sexual partners [34].

Multiple HR-HPV were frequently detected in anal swabs from Bamako’s MSM, mainly in HIV-infected individuals. Multiple anal HR-HPV infection in MSM living in sub-Saharan Africa was previously reported [10, 29, 35, 36]. High rates (91–94%) of multiple anal HPV infections with numerous HPV genotypes ranging from 0 to 18 (mean, 4.8–5.0) were also reported in HIV-positive MSM living outside Africa, such as North Canada and Thailand [37, 38]. In the present series, multiple anal HR-HPV infection was 5.5-times more frequent in HIV-positive than in HIV-negative MSM (univariate and multivariate analyses). Indeed, high-risk sexual behavior, including exclusive sex with other men while being HIV-infected, constitutes a significant cofactor strongly associated with increased risk of multiple anal infections with HR-HPV genotypes [39, 40]. Furthermore, MSM who are HIV-positive have an increased risk of anal HPV infection and anal cancer, and HIV infection is considered to be an independent risk factor strongly associated with an increased risk of contracting HR-HPV anal infection [40, 41]. Thus, HPV prevalence rates of 89–93% have been reported among HIV-positive MSM in a recent systematic review and meta-analysis of 53 studies [42]. The incidence of anal cancer among HIV-positive MSM is more than 80-fold higher than that observed in the general population [43–45]. HIV infection has been shown to increase susceptibility to persistent HPV, increasing the risk of acquiring new HPV infections and reactivation of latent HPV infections [37]. Persons who are at high-risk for HIV acquisition may be at higher risk of HPV infection due to the same high-risk sexual practices [25]. Overall, our observations confirm the close and probably synergistic links between HIV infection, the high prevalence of HR-HPV anal shedding and by extension, the risk of anal cancer in MSM living in sub-Saharan Africa.

Our study had some limitations. Indeed, the recruitment of participants from only the Clinic of the National NGO Soutoura in Bamako focused on the care of key populations as well as the small sample size of our study population, may have introduced selection bias, with particularly elevated HIV prevalence of study MSM and high access to antiretroviral treatment. Thus, the study participants may be not completely representative of the MSM community of the Mali, especially regarding the prevalences of HIV and anal HPV, and the genotypes distribution of anal HPV. Furthermore, some risk factors may have been underestimated in the statistical analyses.

In conclusion, MSM community living in Bamako constitutes a vulnerable population at high-risk for HR-HPV anal infections. MSM in Mali should urgently receive adapted STIs and anal cancer prevention, screening and care, with the implementation of innovative and adapted preventive interventions against HPV infection and associated cancers.

## Data Availability

The datasets supporting the conclusions of this article are included within the article.

## Abbreviations

ACS: American Cancer Society
95% CI: 95% confidence intervals
DNA: Desoxyribonucleic acid
HIV: Human Immunodeficiency virus
HPV: Human Papillomavirus
HR-HPV: High-risk Human Papillomavirus
MSM: Men who have sex with men
MSMW: Men having sex with both men and women
aOR: adjusted Odds ratios
cOR: crude Odds ratios
STI: Sexually transmitted infections
WHO: World health organization.
