# Correction: High Prevalence of Anal Oncogenic Human Papillomavirus Infection in Young Men Who Have Sex with Men Living in Bamako, Mali

**DOI:** 10.1186/s13027-021-00394-z

**Published:** 2021-08-02

**Authors:** Donato Koyalta, Ralph-Sydney Mboumba Bouassa, Almoustapha Issiaka Maiga, Aliou Balde, Jules Bashi Bagendabanga, Almahdy Ag Alinity, David Veyer, Hélène Péré, Laurent Bélec

**Affiliations:** 1 Centre Hospitalo-Universitaire Gabriel Touré, Bamako, Bamako Mali; 2 Faculté des Sciences de la Santé Humaine de N’Djamena, N’Djamena, Chad; 3 Ecole Doctorale Régionale en Infectiologie Tropicale, Franceville, Gabon; 4 Laboratoire de Virologie, Hôpital Européen Georges Pompidou, Assistance Publique-Hôpitaux de Paris, and Université de Paris, Paris, France; 5 Laboratoire du Centre Hospitalo-Universitaire Gabriel Touré, Bamako, Mali; 6 Pierre Louis Institute of Epidemiology and Public Health (IPLESP), Sorbonne University, INSERM, Paris, France; 7 Project Linkages – Family Health International, Bamako, Mali; 8 Clinique Soutoura, Bamako, Mali

**Correction to: Infect Agents Cancer 16, 51 (2021)**

**https://doi.org/10.1186/s13027-021-00385-0**

Following publication of the original article [1], the authors identified an error in the author name of Almoustapha Issiaka Maiga

- The incorrect author name is: Almoustapha Maiga
- The correct author name is: Almoustapha Issiaka Maiga

The author group has been updated above and the original article [1] has been corrected.
